# Inhibition of RUNX1 slows the progression of pulmonary hypertension by targeting CBX5

**DOI:** 10.17305/bb.2024.10720

**Published:** 2024-08-16

**Authors:** Ximiao Ma, Yiqiu Cao, Dongpeng Yang, Zhu Dong, Xiaowu Wang

**Affiliations:** 1 The First School of Clinical Medicine, Southern Medical University, Guangzhou, China; 2 Department of Cardiothoracic Surgery, Central South University Xiangya School of Medicine Affiliated Haikou Hospital, Haikou, China; 3 Department of Cardiovascular Surgery, People’s Liberation Army General Hospital of Southern Theater Command, Guangzhou, China; 4 Department of Cardiac Surgery, Hainan General Hospital, Hainan Affiliated Hospital of Hainan Medical University, Haikou, China; 5 Department of Cardiovascular Surgery, Zhujiang Hospital of Southern Medical University, Guangzhou, China

**Keywords:** Pulmonary hypertension (PH), pulmonary artery smooth muscle cell (PASMC), RUNX1, chromobox 5 (CBX5), ubiquitination

## Abstract

Pulmonary artery smooth muscle cell (PASMC) dysfunction is the central pathogenic mechanism in pulmonary hypertension (PH). This study explored the mechanism of action of RUNX1, a potential therapeutic target for PH, in PASMCs. A PH mouse model was used to investigate the impacts of RUNX1 knockdown on hemodynamics, right ventricular hypertrophy (RVH), and pulmonary artery remodeling (hematoxylin–eosin [H&E] staining). Isolated PASMCs were transfected with RUNX1- or chromobox 5 (CBX5)-related vectors and then subjected to cell function assays. Immunoprecipitation was used to detect molecular binding and ubiquitination. RUNX1 knockdown reduced right ventricular systolic pressure (RVSP), RVH, and pulmonary artery remodeling in mice with PH. Knockdown of RUNX1 or CBX5 suppressed proliferation, invasion, and migration and stimulated apoptosis in PASMCs under hypoxia. RUNX1 enhanced ubiquitin-specific protease 15 (USP15) promoter activity. USP15 bound to CBX5 and reduced CBX5 ubiquitination, thereby promoting CBX5 expression. CBX5 overexpression promoted the proliferation and movement of hypoxic PASMCs with reduced RUNX1 expression and decreased their apoptosis. In conclusion, RUNX1 knockdown inhibits USP15 transcription to promote the ubiquitination and degradation of CBX5, thereby alleviating PH in mice and reducing hypoxia-induced PASMC dysfunction.

## Introduction

Pulmonary hypertension (PH) is a collective term for a range of conditions leading to elevated pressures (> 20 mmHg) in the pulmonary arteries, affecting at least 1% of the world’s population [1]. The prevalence increases by up to 10% in individuals aged over 65 years [2]. The 1-year survival rate for PH is generally 68% or higher [3]. PH is often caused by advanced diseases in the lungs or left heart and more rarely results from chronic emboli or primary vasculopathy [4]. Regardless of its cause, PH is almost always associated with increased mortality [3]. Adverse remodeling of the arterial tree occurs in PH, resulting in elevations of vascular resistance and right ventricular afterload and eventual heart failure [5]. The transformation of pulmonary artery smooth muscle cells (PASMCs) into a hyperproliferative, migratory, invasive, and apoptosis-resistant phenotype is thought to be a key cause of pulmonary vascular remodeling in PH [6]. Therefore, exploring the molecular mechanisms behind the phenotypic switching of PASMCs is critically important for providing therapeutic targets for the treatment of PH.

Runt-related transcription factor 1 (RUNX1) is a master regulator of embryonic development in humans. It is well known for its essential role in developmental hematopoiesis, from the establishment of the hematopoietic system to the maintenance of hematopoietic homeostasis [7]. RUNX1 abnormalities, occurring through either genetic or non-genetic mechanisms, are involved in the development of various hematological malignancies [8]. A recent study shows that inhibition of RUNX1 can repress endothelial-to-hematopoietic transformation and macrophage recruitment and activation in Sugen/hypoxia (SuHx)-induced PH, suggesting the potential of RUNX1 as a therapeutic target for the treatment of PH [9]. However, the molecular mechanism through which RUNX1 exerts its influence in PH remains unexplored.

RUNX1 can interact with SUV39H1, a histone methyltransferase, that methylates H3K9 to provide high-affinity binding sites for proteins of the heterochromatin protein 1 (HP1) family [10, 11]. HP1α, also known as chromobox 5 (CBX5), has been shown to interact with SUV39H1 to repress inflammatory gene expression in vascular smooth muscle cells (VSMCs) [12], but its role in PASMCs and PH remains unknown. CBX protein expression can be controlled through ubiquitination [13]. It has been shown that ubiquitin-specific protease 15 (USP15), a protein-stabilizing deubiquitinase, is upregulated in PH and promotes pulmonary vascular remodeling [14]. RUNX1 can transcriptionally activate deubiquitinase USP9X to promote liver fibrosis [15], but whether there is an interaction between RUNX1 and deubiquitinase USP15 is not known.

We hypothesize that RUNX1 modulates PH progression through its regulatory effects on USP15 and CBX5, offering potential new targets for therapeutic intervention.

## Materials and methods

### Establishment of a PH mouse model

Male C57BL/6 mice (∼8 weeks old, 20–25 g; Hunan SJA Laboratory Animal Co., Ltd., Changsha, Hunan, China) were housed (temperature: 20 ^∘^C–24 ^∘^C; humidity: 55%–65%) and subjected to experiments with the approval of Southern Medical University’s Ethics Committee. The mice were randomly divided into four groups: sham, model, model + sh-NC, and model + sh-RUNX1. Mice in the model group were kept in a normal pressure chamber with 10% O_2_ for 21 days, while those in the sham group were maintained in a normal environment. From the beginning of the modeling process, mice in the model + sh-NC and model + sh-RUNX1 groups were injected with sh-NC and sh-RUNX1 (50 nmol, ip), respectively, every three days for 21 days.

### Assessment of hemodynamics and right ventricular hypertrophy (RVH)

Mice were anesthetized by inhalation of isoflurane (1%–2%, 0.6–1 L/min), followed by the insertion of catheters into the right ventricle and left carotid artery to measure right ventricular systolic pressure (RVSP) (PowerLab 8/35; ADInstruments, AUS). The mice were then sacrificed by cervical dislocation for isolation of their right ventricle (RV), septum (S), and left ventricle (LV). The RV/(LV + S) weight ratio was used to represent the degree of RVH.

### Quantification of pulmonary artery remodeling

Mouse lung tissue sections were stained with hematoxylin–eosin (H&E) for microscopic evaluation of pulmonary arteries with an outer diameter of 25–100 µm. Pulmonary artery wall thickness (WT) vs total thickness (TT, WT/TT) and wall area (WA) vs total area (TA, WA/TA) were analyzed using Image-Pro Plus 6.0 (Media Cybernetics) [16]. Immunohistochemistry was used to detect α-SMA expression in lung tissue.

### Isolation and culture of primary PASMCs

Male C57BL/6 mice (6–8 weeks old, 20–25 g) were anesthetized by inhalation of isoflurane (1%–2%, 0.6–1 L/min), and their lungs were transferred to PBS (10010023; Gibco, New York, NY, USA) with 1% penicillin–streptomycin. After removing surrounding lung tissue, extravascular fascia, and lining endothelial cells, pulmonary arteries were cut and digested in 0.2% collagenase I (17100017; Gibco) for 40 min at 37 ^∘^C. Primary cells were cultured (37 ^∘^C, 5% CO_2_) in DMEM (11965092; Gibco) with 20% FBS (16140071; Gibco) and 1% penicillin–streptomycin. PASMCs at passages 4–6 were harvested. Cells in the normoxia group were cultured at 37 ^∘^C for 24 h in an ordinary incubator containing 21% O_2_, 74% N_2_, and 5% CO_2_. Cells in the hypoxia group were cultured at 37 ^∘^C for 24 h in an incubator with 5% O_2_, 90% N_2_, and 5% CO_2_.

### Cell transfection

RUNX1 shRNA (sh-RUNX1), USP15 overexpression vector (OE-USP15), USP15 shRNA (sh-USP15), CBX5 overexpression vector (OE-CBX5), CBX5 shRNA (sh-CBX5), and negative controls (OE-NC and sh-NC) (all from RiboBio, Guangzhou, China) were transfected into PASMCs using a riboFECT™ CP transfection kit (RiboBio). Relevant gene expression was measured after 48 h.

### Cell counting kit (CCK)-8 assay

PASMCs were seeded in a 96-well plate (1000 cells/well). Hypoxia was induced the next day. On the third day, CCK-8 reagent (10 µL, C0037; Beyotime, Shanghai, China) was used to detect cell viability.

### Transwell assay

Cells (5000/well) and DMEM (100 µL/well) were added to the upper compartments of a Transwell plate (8 µm; Corning, NY, USA), and DMEM containing 10% FBS was added to the lower compartments. After 24 h of culture under normoxic/hypoxic conditions, cells were fixed in 4% paraformaldehyde, stained with crystal violet, and imaged using a standard microscope (Leica DMi8; Germany).

### Flow cytometry

At 80% confluence, 1 × 10^6^ cells were taken for Annexin V apoptosis detection (88-8005-74; eBioscience, New York, NY, USA). Briefly, the cells were suspended in 1X Annexin buffer, incubated with 5 µL of Annexin V-FITC, washed, resuspended in 300 µL of 1X Annexin buffer, and analyzed using a flow cytometer (Guava^®^ easyCyte 12; Millipore, USA).

### Scratch assay

Cells were seeded in a 6-well plate (2 × 10^5^ cells/well) for 24 h and then cultured without serum for another 24 h. The cell monolayers were scratched with a 200-µL pipette tip and further cultured for 48 h. ImageJ was used to measure the distances that the cells migrated from the edges of the scratches.

### Chromatin immunoprecipitation (ChIP)

Experiments were performed according to the instructions of a ChIP kit (26156; Thermo Fisher Scientific, New York, NY, USA). After crosslinking, cells were lysed and chromatin was sonicated into 300–1000-bp fragments. Input (50 µL of supernatant) was preserved at −20 ^∘^C, while 500 µL of supernatant in 1 mL of hybridization buffer was incubated with 50 pmol of anti-IgG or anti-RUNX1 antibody (1:800, ab92336; Abcam, Cambridge, UK) at 37 ^∘^C for 4 h. The immune complexes were rotated with washed streptavidin beads at 37 ^∘^C for 0.5 h. The bead complexes were collected by centrifugation, washed five times, treated with 50 µL of DNA elution buffer plus RNase A (100 µg/mL) and RNase H (0.1 U/µL) at 37 ^∘^C for 0.5 h, boiled (100 ^∘^C) with 10 µL of 5X loading buffer for 10 min, and finally centrifuged. The supernatant was used for RT-qPCR detection.

### Co-immunoprecipitation (co-IP)

The proteins obtained from cell lysates were incubated with an anti-CBX5 (1:1000, ab109028) or anti-IgG (1:1000, ab172730) antibody at 4 ^∘^C overnight and then rotated with protein G/A beads at 4 ^∘^C for 3–5 h. The mixture was centrifuged at 1000 × *g* for 5 min at 4 ^∘^C. The immune precipitate was washed three times with a washing buffer (50-mM Tris-HCl/pH 7.4, 100-mM NaCl, 5-mM CaCl_2_, 5-mM MgCl_2_, and 0.1% Nonidet P-40) and resuspended in 1X SDS-PAGE loading buffer for western blotting with an anti-USP15 (1:800, ab71713) or anti-ubiquitin (1:1000, ab134953) antibody. All antibodies were from Abcam.

### Luciferase reporter assay

pGL3-Basic was inserted with a USP15 promoter sequence (RiboBio) and co-transfected with sh-RUNX1 into PASMCs for 48 h. Renilla luciferase activity was measured using the Dual-Glo Luciferase Assay System (Promega) and normalized to the internal control firefly luciferase activity.

### Cycloheximide (CHX) pulse-chase assay

The effect of USP15 on CBX protein half-life was examined using a CHX pulse-chase assay combined with a standard protein synthesis inhibitor. PASMCs transfected with USP15 or a control vector were treated with 60-µg/mL CHX for different durations. Finally, proteins were extracted and analyzed using western blotting.

### RT-qPCR

Total RNA was extracted from cells/tissues using 1 mL of TRIzol (Thermo Fisher Scientific) and reverse-transcribed with M-MLV reverse transcriptase and random primers. PCR experiments were performed using a Premix Ex Taq™ II kit (Takara, Dalian, China) and an ABI7500 instrument (Applied Biosystems, Shanghai, China), with GAPDH as the internal reference for mRNA (see Table 1 for the PCR primers used). Data were analyzed using the 2^-ΔΔCt^ method [17].

**Table 1:** Primer sequences used in this study

**Primers**	**Sequences**
RUNX1-F	TGTTGGTCATGGATGGGGTG
RUNX1-R	ACTTGGGCTCTAGTGGTCTCA
CBX5-F	ACTGGCAGCCGTACTAGACT
CBX5-R	CGTGAGCTGCAATCCTGTTG
USP15-F	GGCCTGGCTGTTCATTGTTT
USP15-R	TGTGATGTAATTGGCCACATTTTT
GAPDH-F	GTGGCTGGCTCAGAAAAAGG
GAPDH-R	GGGGAGATTCAGTGTGGTGG

F: Forward primer; R: Reverse primer.

### Western blotting

The total protein concentration of cell/tissue lysates was determined using a BCA kit (23227; Thermo Fisher Scientific). After electrophoresis, separated proteins were transferred onto a membrane. After blocking in 5% (w/v) non-fat powdered milk in 1X PBS for 1 h at ambient temperature, the membrane was incubated with primary antibodies (RUNX1, 1:1000, ab92336; CBX5, 1:800, ab109028; USP15, 1:800, ab71713; β-actin, 1:1500, ab8226; all from Abcam) overnight at 4 ^∘^C and then with a secondary antibody (1:3000, ab150077; Abcam) for 1 h at ambient temperature. Blot images were captured using the BioSpectrum Imaging System (UVP, USA).

### Statistical analysis

Data were analyzed using GraphPad Prism 8.0, with all quantitative data expressed as mean ± SEM. After the Shapiro–Wilk test for normality, parameters of three or more groups were compared by one-way ANOVA, while differences between two groups were analyzed using Student’s *t*-test. The Bonferroni test was applied for post-hoc comparisons. A *P* value less than 0.05 was considered statistically significant.

## Results

### RUNX1 knockdown alleviates hypoxia-induced PH in mice

**Figure 1.**
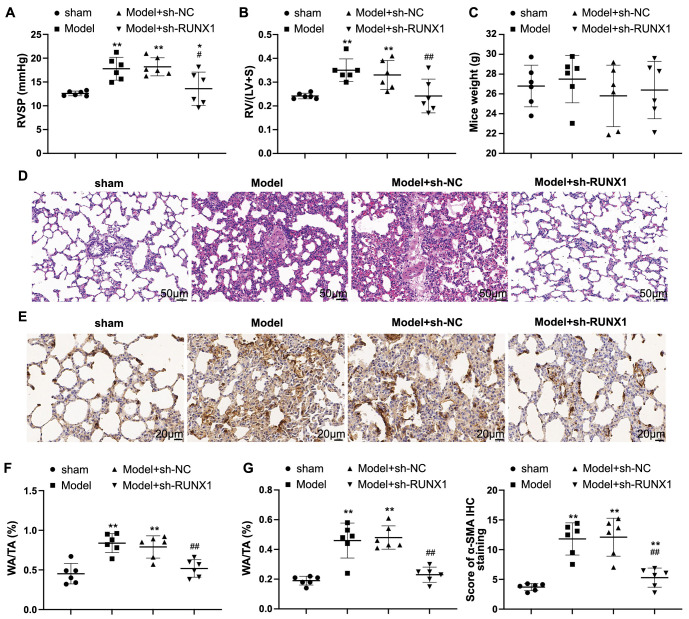
**RUNX1 knockdown alleviates PH in mice.** A hypoxia-induced PH mouse model was established: (A) RVSP; (B) RVH, measured as the ratio of right ventricle (RV) weight to left ventricle (LV) + septum (S) weight [RV/(LV + S)]; (C) Body weight of mice at the end of the experiment; (D) H&E staining to evaluate pulmonary vascular remodeling; (E) Immunohistochemistry to detect α-SMA expression in lung tissue; (F) Ratio of pulmonary artery wall area (WA) to total area (TA, WA/TA); (G) Ratio of arterial wall thickness (WT) to total thickness (TT, WT/TT). *N* ═ 6; *P < 0.05 and **P < 0.01 vs the sham group; ^#^*P* < 0.05 and ^##^*P* < 0.01 vs the model group. RVH: Right ventricular hypertrophy; RVSP: Right ventricular systolic pressure; PH: Pulmonary hypertension; RVSP: Right ventricular systolic pressure; H&E: Hematoxylin–eosin.

A study shows that targeted inhibition of RUNX1 may serve as a new treatment modality for PH [9], but the mechanism through which RUNX1 exerts its influence has not yet been studied. In this study, we first examined the effect of knocking down RUNX1 on hypoxia-induced PH in mice. Hemodynamic results showed that RVSP and RV/(LV + S) increased in the model group (vs the sham group) but decreased in the model + sh-RUNX1 group (vs the model + sh-NC group) (Figure 1A and 1B, *P* < 0.05). There was no significant change in mouse body weight between these groups (Figure 1C, *P* > 0.05). H&E staining revealed a reduction in hypoxia-induced pulmonary artery remodeling in the model + sh-RUNX1 group (vs the model + sh-NC group) (Figure 1D). Immunohistochemistry showed that the percentage of α-SMA-positive cells was reduced in the model + sh-RUNX1 group compared to the model + sh-NC group (Figure 1E, *P* < 0.05). The model + sh-RUNX1 group had lower WA/TA and WT/TT ratios (%) than the model + sh-NC group (Figure 1F and 1G, *P* < 0.05). The above data indicate that knocking down RUNX1 can alleviate hypoxia-induced PH.

### RUNX1 knockdown reduces hypoxia-induced PASMC dysfunction

**Figure 2.**
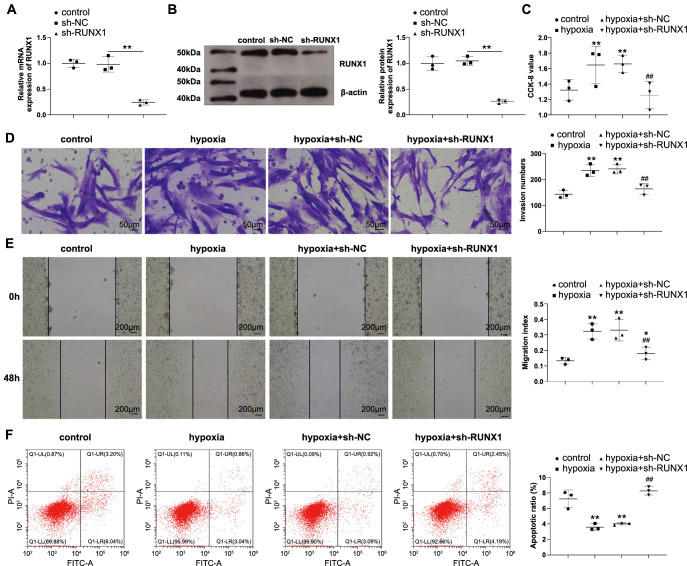
**RUNX1 knockdown alleviates hypoxia-induced PASMC dysfunction.** The regulation of RUNX1 on hypoxic PASMCs was investigated: RT-qPCR (A) and western blotting (B) were used to detect RUNX1 mRNA and protein expression; (C) CCK-8 was used to detect cell proliferation; (D) Transwell assay was performed to detect cell invasion; (E) Scratch assay was performed to detect cell migration; (F) Flow cytometry was used to detect cell apoptosis. *N* ═ 3; *P < 0.05 and **P < 0.01 vs the control group; ^#^*P* < 0.05 and ^##^*P* < 0.01 vs the hypoxia group. PASMC: Pulmonary artery smooth muscle cell; CCK: Cell counting kit; RT-qPCR: Reverse transcription-quantitative polymerase chain reaction.

To detect the influence of RUNX1 on hypoxic PASMCs, we knocked down RUNX1 levels in PASMCs by sh-RUNX1 transfection (Figure 2A and 2B, *P* < 0.05 vs sh-NC) and cultured the cells under hypoxic conditions. RUNX1 knockdown suppressed hypoxia-induced proliferation (Figure 2C, *P* < 0.05), invasion (Figure 2D, *P* < 0.05), and migration (Figure 2E, *P* < 0.05) of PASMCs and sensitized hypoxic PASMCs to apoptosis (Figure 2F, *P* < 0.05).

### CBX5 promotes hypoxia-induced PASMC proliferation and migration

**Figure 3.**
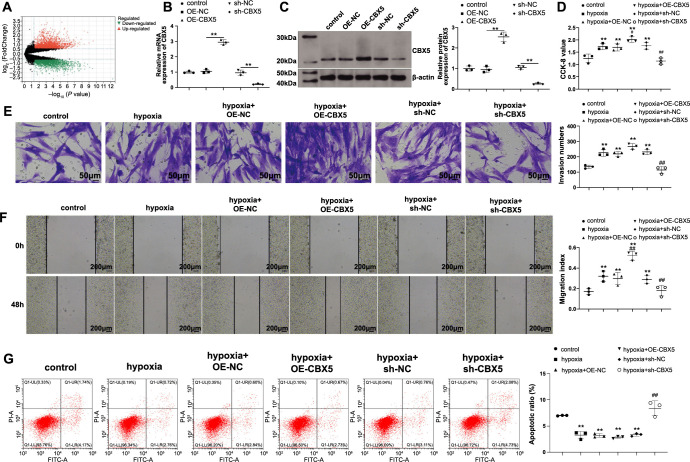
**CBX5 promotes hypoxia-induced PASMC dysfunction.** The regulation of CBX5 on hypoxic PASMCs was investigated: (A) Differential gene expression data were obtained from the GSE15197 dataset; RT-qPCR (B) and western blotting (C) were used to detect CBX5 mRNA and protein expression; (D) CCK-8 was used to detect cell proliferation; (E) Transwell assay was performed to detect cell invasion; (F) Scratch assay was performed to detect cell migration; (G) Flow cytometry was used to detect cell apoptosis. *N* ═ 3; *P < 0.05 and **P < 0.01 vs the control group; ^#^*P* < 0.05 and ^##^*P* < 0.01 vs the hypoxia group. PASMC: Pulmonary artery smooth muscle cell; CBX5: Chromobox 5; CCK: Cell counting kit; RT-qPCR: Reverse transcription-quantitative polymerase chain reaction.

To identify downstream targets of RUNX1, we screened datasets of gene expression in patients with PH from the GEO database and selected the GSE15197 dataset, which includes frozen fresh lung tissue specimens from 18 patients with PH and 13 healthy controls. The R language limma package was used to analyze gene expression in the lung tissues. Genes with |LogFC| > 1 and *P* < 0.05 were considered differentially expressed, and CBX5 was found to be upregulated in patients with PH (Figure 3A). CBX5 is a potential driver of fibroblast activation in pulmonary fibrosis [18], but its role in PH has not been studied.

To investigate the role of CBX5 in PH, we transfected CBX5-related vectors into PASMCs. OE-CBX5 transfection successfully upregulated CBX5 levels, whereas sh-CBX5 transfection successfully downregulated CBX5 levels (Figure 3B and 3C, *P* < 0.05 vs NC). Transfected cells were cultured under hypoxic conditions and then subjected to detection of phenotypic changes. Hypoxic PASMCs overexpressing CBX5 showed improvements in proliferative and migratory abilities; CBX5 knockdown inhibited the proliferation, invasion, and migration of hypoxic PASMCs and stimulated their apoptosis (Figure 3D–3G, *P* < 0.05). These results demonstrate that CBX5 promotes proliferation, invasion, and migration and suppresses apoptosis in PASMCs under hypoxia.

### RUNX1 modulates CBX5 expression via USP15

**Figure 4.**
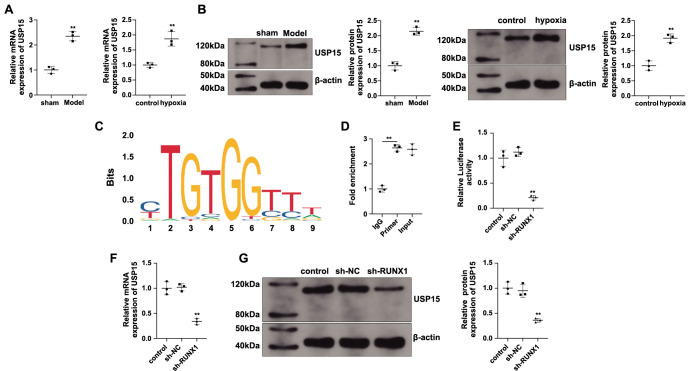
**RUNX1 regulates USP15 transcription.** The regulatory effect of RUNX1 on USP15 was investigated: RT-qPCR (A) and western blotting (B) were used to detect USP15 mRNA and protein expression; (C) The JASPAR database showed a RUNX1-binding motif in the USP15 promoter; (D) ChIP was used to detect the binding of RUNX1 to the USP15 promoter; (E) Luciferase reporter assay was performed to analyze the influence of RUNX1 on USP15 promoter activity; RT-qPCR (F) and western blotting (G) were used to detect the regulation of USP15 mRNA and protein expression by RUNX1 knockdown. *N* ═ 3; **P < 0.01. USP15: Ubiquitin-specific protease 15; ChIP: Chromatin immunoprecipitation.

USP15 is highly expressed in patients with PH, as well as in mouse and rat models of PH [14]. Similarly, we found that USP15 was upregulated in hypoxic PASMCs and in mice with hypoxia-induced PH (Figure 4A and 4B, *P* < 0.05). The JASPAR database (https://jaspar.elixir.no/) shows that RUNX1, as a transcription factor, can directly bind to USP15, and the binding motif is displayed in Figure 4C. ChIP experiments demonstrated that RUNX1 strongly binds to the promoter of USP15 (Figure 4D, *P* < 0.01 vs IgG). To observe the influence of RUNX1 on the USP15 promoter, we constructed a luciferase reporter vector inserted with a USP15 promoter fragment. In PASMCs, RUNX1 knockdown significantly inhibited the luciferase activity of the USP15 promoter construct (Figure 4E, *P* < 0.01). Moreover, USP15 expression was inhibited in PASMCs transfected with sh-RUNX1 (Figure 4F and 4G, *P* < 0.01 vs sh-NC). Collectively, these results indicate that RUNX1 regulates USP15 promoter activity by binding to the USP15 promoter, thereby activating USP15 expression.

**Figure 5.**
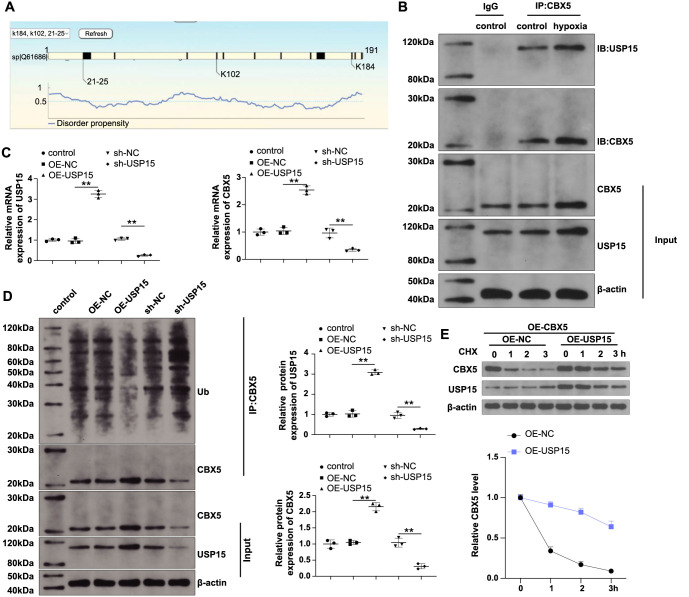
**USP15 regulates CBX5 ubiquitination.** The regulation of USP15 on CBX5 was investigated: (A) The GPS6.0 database predicted CBX5 ubiquitination sites; (B) Co-IP was used to detect the binding of CBX5 to USP15; (C) RT-qPCR was used to detect USP15 mRNA and CBX5 mRNA levels; (D) Western blotting was used to detect USP15 protein and CBX5 protein levels, as well as CBX5 ubiquitination; (E) CHX was used to detect the effect of USP15 on CBX5 protein stability. *N* ═ 3; **P < 0.01. CBX5: Chromobox 5; USP15: Ubiquitin-specific protease 15; CHX: Cycloheximide.

USP15 is a deubiquitinating enzyme that stabilizes proteins by regulating their deubiquitination. The GPS6.0 database (http://gps.biocuckoo.cn/index.php) shows that there are multiple ubiquitination modification sites in the CBX5 amino acid sequence (Figure 5A). To determine whether USP15 regulates CBX5 ubiquitination and, thus, affects its expression, we first used an anti-CBX5 antibody to precipitate CBX5 in control and hypoxic PASMCs and found USP15 in those immune precipitates (Figure 5B), indicating that USP15 can bind to CBX5 in PASMCs. Next, we transfected USP15-related vectors into PASMCs. OE-USP15 transfection increased USP15 and CBX5 expression, whereas sh-USP15 transfection decreased USP15 and CBX5 expression (Figure 5C and 5D, *P* < 0.01 vs NC). Co-IP experiments further showed that USP15 overexpression reduced the ubiquitination of CBX5, whereas USP15 knockdown promoted the ubiquitination of CBX5 (Figure 5D). Finally, a CHX chase assay was performed to assess the effect of USP15 on CBX5 stability. OE-USP15 transfection significantly stabilized CBX5 protein levels, whereas CBX5 protein was obviously degraded following OE-NC transfection (Figure 5E). Taken together, these results indicate that USP15 can reduce the ubiquitination of CBX5 protein, thereby enhancing its stability.

### RUNX1 regulates CBX5 to stimulate hypoxia-induced PASMC dysfunction

**Figure 6.**
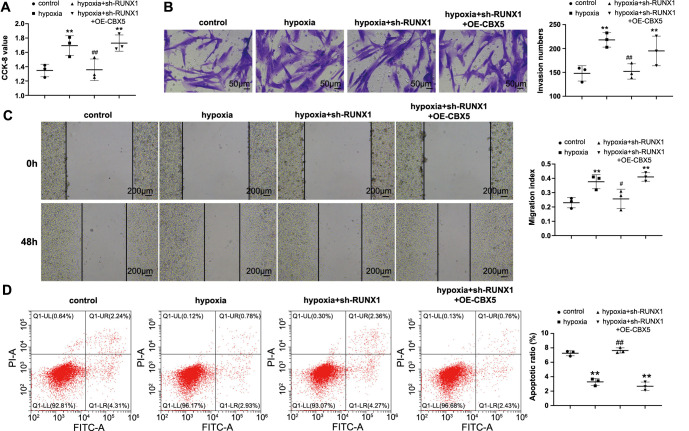
**RUNX1 regulates CBX5 to promote hypoxia-induced PASMC dysfunction.** PASMCs were transfected with sh-RUNX1 and OE-CBX5: (A) CCK-8 was used to detect cell proliferation; (B) Transwell assay was performed to detect cell invasion; (C) Scratch assay was performed to detect cell migration; (D) Flow cytometry was used to detect cell apoptosis. *N* ═ 3; *P < 0.05 and **P < 0.01 vs the control group; ^#^*P* < 0.05 and ^##^*P* < 0.01 vs the hypoxia group. PASMC: Pulmonary artery smooth muscle cell; CBX5: Chromobox 5; CCK: Cell counting kit.

To confirm whether RUNX1 regulates hypoxia-induced PASMC dysfunction by modulating CBX5 expression, we transfected sh-RUNX1 together with OE-CBX5 into PASMCs. Compared with the hypoxia + sh-RUNX1 group, the hypoxia + sh-RUNX1 + OE-CBX5 group exhibited higher proliferative, invasive, and migratory abilities and a lower apoptosis rate (Figure 6A–6D, *P* < 0.05). From the above, it is evident that RUNX1 mediates the proliferation, movement, and apoptosis of PASMCs under hypoxia by promoting CBX5 expression.

## Discussion

Pulmonary vascular remodeling is the key structural alteration in PH [19]. This process is characterized by smooth muscle cell (SMC) coating of normally non-muscularized distal pulmonary arterioles—involving migration and robust proliferation of SMCs—and hypertrophy of SMCs in more proximal arteries [20]. This study investigated PH-related molecular interactions in PASMCs and found: 1) RUNX1 promotes hypoxia-induced PASMC dysfunction and PH; 2) RUNX1 transcriptionally activates the expression of USP15; 3) USP15 reduces CBX5 ubiquitination and promotes CBX5 expression; and 4) RUNX1 regulates PASMC function through the USP15/CBX5 axis.

RUNX1 is a master developmental regulator essential for hematopoietic cell differentiation and normal hematopoiesis [21]. RUNX1 mutations are observed in various types of leukemia, as well as other hematological diseases [22]. RUNX1 abnormalities have also been found to be associated with non-hematological diseases, such as cardiovascular disease [23], osteoarthritis [24], and acute lung injury [25]. Moreover, by administering a RUNX1 inhibitor or deleting the Runx1 gene, Jeong et al. [9] found that RUNX1 is required for endothelial-to-hematopoietic transformation and macrophage recruitment and activation in SuHx-induced PH. Endothelial dysfunction and subsequent inflammation contribute to pulmonary vascular remodeling in PH [26]. The regulation of RUNX1 on endothelial progenitor cell fate suggests its crucial role in pulmonary vascular remodeling. The phenotypic transformation of PASMCs is another key cause of pulmonary vascular remodeling in PH [6]. RUNX1 has previously been shown to induce serine elastase activity, which is essential for structural remodeling, in PASMCs [27, 28], suggesting that RUNX1 can also regulate pulmonary artery remodeling through its effect on the PASMC function. This study found that RUNX1 knockdown reduced RVSP, RVH, and pulmonary artery remodeling in mice with hypoxia-induced PH. RUNX1 knockdown also suppressed hypoxia-induced proliferation, invasion, and migration of PASMCs, and sensitized hypoxic PASMCs to apoptosis. Moreover, RUNX1 was found to have a binding site in the USP15 promoter. By binding to the USP15 promoter, RUNX1 increased USP15 transcription and promoted USP15 expression in PASMCs.

USP15 belongs to the USP family of deubiquitinating enzymes that mediate the reverse process of ubiquitination, called deubiquitination. Ubiquitination is a post-translational modification involving the covalent attachment of ubiquitin to target proteins, leading to either proteasome-mediated degradation or proteasome-independent regulation of these proteins [29]. Deubiquitinases are specialized proteases that reverse ubiquitination by removing ubiquitin from substrates or cleaving ubiquitin chains [30]. Abnormal expression of USP15 has been identified in various cancers and other diseases [31]. Wu et al. [14] found that USP15 promotes PASMC proliferation and migration by inhibiting K48-linked ubiquitination of YAP1 and interacting with TAZ, contributing to pulmonary vascular remodeling in PH. Moreover, USP15 upregulation resulting from FUNDC1 deficiency promotes TNF-α-induced pulmonary artery endothelial cell proliferation by increasing Drp1 deubiquitination to regulate mitochondrial dynamics [32]. These findings show that USP15 acts in both endothelial cells and SMCs to regulate pulmonary vascular remodeling. RUNX1 may promote PASMC proliferation and migration by interacting with USP15. This study found that USP15 was upregulated in hypoxic PASMCs and in mice with hypoxia-induced PH. More importantly, USP15 reduced the ubiquitination of CBX5 protein and promoted its expression in PASMCs.

CBX5 is a chromobox protein of the HP1 family and a methyl reader that interprets H3K9me2/3 marks mediated by H3K9 methyltransferases (i.e., SUV39H1 and SUV39H2) [33]. CBX5 contains two highly conserved domains: an N-terminal chromodomain, responsible for recognizing heterochromatin marks, and a C-terminal chromoshadow domain, responsible for mediating homo- and heterodimerization [34]. Various functions have been described for CBX5, including gene silencing by heterochromatin formation, heterochromatin maintenance, telomere capping, DNA repair, and gene expression regulation [35]. Recent studies on the associations between CBX5 and diseases have found that CBX5 regulates tumor cell growth and apoptosis in various cancers [36–38], periodontal ligament cell apoptosis in periodontitis [39], and fibroblast activation in lung fibrosis [18]. However, the function of CBX5 in PH remains unclear. A previous study shows that the recruitment of SUV39H1 and CBX5 to inflammatory genes is reduced in diabetic VSMCs [12], suggesting that CBX5 may be dysregulated in VSMCs under disease conditions. In addition, CBX5 overexpression can promote the differentiation and angiogenic activity of endothelial progenitor cells [40]. These findings indicate that CBX5 plays a significant role in regulating endothelial and SMC function. Consequently, CBX5 may regulate pulmonary vascular remodeling in PH. The influence of the RUNX1/USP15 axis on the PASMC function may result from USP15’s interaction with CBX5. In this study, CBX5 promoted proliferation, invasion, and migration, while suppressing apoptosis in hypoxic PASMCs. Moreover, CBX5 overexpression eliminated RUNX1 knockdown-induced inhibition of PASMC dysfunction under hypoxia.

## Conclusion

In summary, RUNX1 stimulates hypoxia-induced PASMC dysfunction by increasing USP15 transcription to promote the deubiquitination and expression of CBX5. This work uncovers a novel molecular mechanism underlying the development of PH and opens a new avenue for developing targeted treatments for this severe vascular condition. RUNX1 can be targeted using approaches such as specific inhibitors and tissue-specific gene deletion, thereby affecting the downstream USP15/CBX5 axis to alleviate PH. Moreover, the expression of the RUNX1/USP15/CBX5 axis may be utilized to diagnose PH and predict disease severity.

## Data Availability

The datasets used or analyzed during the current study are available from the corresponding author on reasonable request.
